# Books

**Published:** 1883-02

**Authors:** 


### Books.

RHEUMATISM, GOUT AND SOME ALLIED DISORDERS. By Morris Longstreth, M.D. New York: William Wood & Co. 1882. Wood's Library of Standard Medical Authors.

This volume consists of quite a full, and no doubt very faithful history of the subjects to which it is devoted. With, perhaps, unnecessary prolixity divers theories and opinions, collated from a variety of sources, are presented, respecting the causes, clinical history, pathology, complications, diagnosis, prognosis and treatment of acute rheumatism; and, more briefly, we find chronic articular rheumatism, gonorrhœal rheumatism and gout severally considered in the last three chapters of the book.

Under the head of complications, or extra-articular localizations the author discusses what were formerly considered metastases of the rheumatic inflammation from the joints to other parts and to different organs of the body, but what are now generally described as complications, and what he thinks it would be more philosophical to regard as multiple localization of the rheumatic condition.

These complications or localizations occur, according to the writer, in order of frequency in the muscular, circulatory, respiratory, cerebro-spinal, digestive and urino-genital systems.

As to treatment of rheumatism by drugs, the alkalies, bromide of potassium and salicylic acid or salicylate of soda are specially commended. Guaicum is useless, so is colchicum, but the latter is valuable in gout.

In reference to the *modus operandi* of the salicyl treatment, the writer gives expression to the following practical and very sensible paragraph. He says: "It seems possible to explain the curative effects of the salicyl compounds without calling up the presence of unknown organisms with the prospect of rapid death before them. It is possible to think of disease, not as a foreign entity which takes up its residence in our tissues, producing disturbance and destruction by new and foreign methods. It seems much more likely that disease is a misdirection given to our normal functions by agents other than minute organisms. We have been accustomed to think of disease as changed, depressed, or exaggerated function, but still always to be recognized as function, and not to think of medicine as a ferret sent in to hunt out and destroy an interloper in our bodies. We have always sought to think of medicine as guiding the formative and retrograde processes of nutrition according to the normal physiological methods, and by changing the nervous action or influence to alter the blood supply, which is the fountain of nutritive activity, normal or pathological."

The book will repay perusal, albeit it contains little or nothing not already known, and it will be serviceable, no doubt, in the series, but the style of the writer—the composition—is susceptible of great and needful improvement.

---

The Diseases of the Liver, With and Without Jaundice, with the Special Application of Physiological Chemistry to their Diagnosis and Treatment. By George Harley, M.D., F.R.S. Illustrated by colored plates and wood engravings. Philadelphia: P. Blakiston, Son & Co. 1883. Pp. 751. Price, cloth, $5.00.

This comprehensive treatise is the outgrowth of a monograph, by the author, on Jaundice, which appeared in England twenty years ago, and it is published in this country simultaneously with the first English edition.

The authors lay down the proposition that a knowledge of animal organization, important though it be, is yet less indispensable to the physician than a knowledge of healthy function, for it is the latter which more especially elucidates the dark problems of life. It is the latter which proves the golden key to the comprehension of disease, and he forthwith proceeds to elucidate some of these dark problems, and to describe, at length, the healthy functions of that wonderful and important organ, the liver.

He holds that "biliverdin is nothing more or less than oxidized blood hæmatin, which the liver does not form, but only extracts from the circulation during the passage of the blood through the hepatic capillaries."

"A crucial test" he says "for all spurious forms of jaundice is a very simple one—namely, a naked eye inspection of the fæces. If the stools be pipe clay colored, the case may be at once put down as one of jaundice. If, however, the stools are dark colored, that circumstance itself does not negative the idea of jaundice; for the dark color of the stools may be due not to the presence in them of bile, but of blood. Or it may be due to medicine—for all metallic remedies whose sulphurets are of a dark color turn black in cases of true jaundice, from the sulphureted hydrogen disengaged during the putrid fermentation of the non-bilifed food in the intestinal canal combining with the metalic remedies, such as mercury, iron, bismuth, etc., and forming black sulphurets, which, mixing with the pipeclay-colored fæces of jaundice, give them the appearance of being colored with bile, and lead to an error in diagnosis."

The practice of relying upon the healing powers of nature in the management of live diseases is deplored and these diseases are asserted to be as easy of diagnosis and as susceptible of properly directed treatment as disease of other organs—as the heart, lungs or kidneys.

Of the appropriate remedies in these disorders mercury is considered invaluable, not only on account of its power of expelling the stored up bile, but in certain cases of diseased liver, where the biliary secretion is retarded, or even arrested, in consequence of a congested condition of the tissues of the liver, mercury has a powerful, though only an indirect, effect in restoring the biliary secretion."

Mineral acids and alkalies—the former to be taken after, and the latter in small quantity, before food—are classed as undoubted hepatic stimulants—that is they increase the secretion of bile. Chloride of ammonium, in doses of twenty to thirty grains, is regarded as entitled to special mention on account of its value in hepatic congestion.

Yellow fever is declared to be a disease of the liver, and is treated under two forms—"specific yellow fever"

and "malarial yellow fever." The term "yellow fever," our author declares, "ought to be expunged from our nosology as being both clinically and pathologically an incorrect definition of the morbid condition the name is intended to convey, and the word 'jaundice' should be substituted," as "contagious jaundice" and "malarial jaundice"!

This is a distinction ; is it a difference?

The author is evidently a man of very clear convictions, and he expresses these convictions in a very forcible manner. In some instances his language is strong—unnecessarily so—and his assertions dogmatic—decidedly so. Yet the honesty of his opinions is above question, and interest in his work is not impaired by his positive style.

It is an entertaining, a very useful and instructive book. It is deserving of a generous reception, and no doubt it will be cordially received.

If it is true that all the pleasures of life are dependent upon the fidelity of the liver, this book should be read and pondered by everybody.

As the author considers time of quite as much value to the professional as to the mercantile man, he has been very considerate in the arrangement of his work, and has managed to condense what he has to say about liver diseases into 750 large pages.

The printing is well done, and the binding is neat and substantial. Two excellent chromo-lithographic plates, and thirty-six wood-cuts, illustrative of the text, adorn the volume.

---

THE DIAGNOSIS AND TREATMENT OF DISEASES OF THE EYE. By Henry W. Williams, A.M., M.D. Boston: Houghton, Mifflin & Co. 1881. Pp 464.

The object of the author, in the preparation of this volume, has been to make a book which would be a practical guide in the study of the diseases of the eye, their diagnosis and treatment, serviceable alike to the general practitioner and to the medical student.

In a chapter on general principles of treatment we note the following sensible suggestions: "Astringent stimulating or sedative solutions are especially serviceable in inflammations of the conjunctival mucous membrane, characterized by smarting or itching sensations rather than by deep-seated aching pain. When the pain assumes the latter form it is generally an indication for the disuse of these means; which are valueless, if not injurious, in inflammations of other than the external envelope. Solutions of atropia and of pilocarpine, to act especially upon the iris, have largely and conveniently taken the place of the extracts of belladonna, etc., formerly employed. Atropia should not be used for conjunctival affections, as is frequently done, as it increases the photophobia.

Solid crayons afford a useful means for obtaining an occasional greater astringent or stimulant effect than that of collyria.

Local depletion should be by leeches applied to the temple and not too near the eye, but is less relied upon than formerly. Counter-irritation in the form of blisters and setons is now rarely resorted to, and is a relic of barber surgery.

Poultices are admissible only in inflammations of the appendages of the eye, and not allowable in diseases of the eye itself, except in suppurative inflammation of the entire globe. In other cases they are dangerous, as being liable to induce or augment corneal ulceration.

A solution of borax, ten grains to the ounce of camphor water, is recommended as preferable to the stronger astringents in the milder form of conjunctivitis, and in corneal affections it is invaluable.

Eserine and pelocarpine are warmly indorsed in certain affections. Their action, as is well understood, being the reverse of that of atropia, and causing contraction of the pupil.

The conspicuous absence of secret signs and special hieroglyphics which some specialists are so prone to affect is not among the least pleasing features of the book, for, while these mystic abbreviations may indicate profound erudition and highly scientific proclivities upon the part of the writers of special treatises, they are, beyond doubt, unmitigating nuisances to the general reader who may be called upon to decipher them, and who has claims upon his attention of more importance than the tedious translation of short-hand figures of speech.

The treatise of Professor Williams is well illustrated, containing several handsome colored plates and the usual test-type employed for determining the degree and force of vision. It will, we believe, prove a useful book to the profession.

---

LEGAL MEDICINE. By Charles Meynott Tidy, M.B., F.C.S. Vol. I, II. New York: William Wood & Co. 1882. Wood's Library of Standard Medical Authors.

Medical Jurisprudence is one of the most fascinating of all the various departments of the medical sciences; and turning from treatises upon pathology, therapeutics, symptomatology, divers diseases, etc., to a well-written and carefully prepared work upon forensic medicine is as refreshing as the perusal of a pleasing romance after studious and wearying application to some profound study in history or the more occult works of general literature.

The two volumes before us are by no means the least interesting or useful of these valuable annual series.

The subject to which they relate is one in which every medical witness—and this means almost every medical practitioner, at one time or another—is deeply concerned; for a becoming deportment and a ready knowledge are greatly to be desired under the often trying circumstances surrounding the witness stand. These points must of necessity be learned from books, as the experience of any one person will scarcely prove sufficient to save him from the many embarrassing and annoying quick-sands which not infrequently surround the medical witness in a temple of justice. In this work he will find many valuable hints and much suggestive information.

"There is," says the author, "a scientific certainty which only the coward treats as uncertainty, and there is an uncertainty which only the boldness of ignorance ignores." These extremes should be watchfully avoided.

The duty of thorough preparation before giving evidence is urged as of great importance, and the necessity of using only plain English in the witness box is very properly insisted upon. The shallow pedant who seeks to impress others with his grandeur and his learning by the employment of gradiloquent and technical expressions when giving evidence for the benefit of an average jury is a fit subject for ridicule, which he rarely escapes, and generally, by his pretentious style, deceives nobody more than himself.

The value of apparent trifles and their important bearing ofttimes upon vital issues is here taught. Illustrative cases, in order to avoid too frequent interruption in the text, are grouped at the end of the several chapters. A copious and excellent index will be found appended to the second volume. Altogether the book is one that may be read and re-read with profit by every member of the class for whom it is intended.

---

LECTURES ON DIEASES OF THE NERVOUS SYSTEM, ESPECIALLY IN WOMEN. By S. Weir Mitchell, M.D. Philadelphia: Henry C. Lea's Son & Co. 1881. Pp. 238.

The author claims that the lectures which compose this little volume deal chiefly with some of the rarer maladies of women. Many of them, he says, are original studies of well-known diseases, others relate to studies hitherto slighted by, or almost unknown to, medical literature. The lectures deal with such subjects as hysterical paralysis; hysterical ataxia, and paresis; mimicry of diseases; rare spasmodic affections; tremor; chorea; disorders of sleep; respiratory and vaso-motor disorders; hysterical aphonia; digestive disorders of hysteria; and the treatment of obstinate cases of nervous exhaustion and hysteria by seclusion, rest, massage, electricity, and full feeding.

In regard to the general treatment of the class of disorders to which these interesting lectures relate, the author very earnestly says: "I have some belief in the occasional value of induction-currents in hystero-palsies, but, as to the direct good to be had out of the drugs on which men once relied in the treatment of this disease, I

have said nothing, because, except to condemn, I had nothing to say, and because I believe that the numberless remedies for hysteria to be found in the books will be swept by another generation into the limbo provided for drugs with decayed reputations; but in thus expressing myself I do not mean to say that no drugs have an indirect value. What you have to do is to rectify with care positive uterine troubles, to treat defects of nutrition, to relieve the anæmia so apt to exist in hysteria, to see that every function is well cared for, and last, not least, to learn what need there is to alter the moral surroundings of your patient, and then with kind and patient care, and an unbending will, to bring about the changes she may seem to require. * * * * * *

The treatment to which in these pages I so many times refer, consists in an effort to lift the health of patients to a higher plane by the use of seclusion, which cuts off excitement and foolish sympathy; by rest, so complete as to exclude all causes of tire; by massage, which substitutes passive exercise for exertion; and by electrical muscular excitation, which acts in a somewhat similar manner to massage, and with it, by depriving rest in bed of its essential evils, leaves only its good. These means enable us to over-feed our patients, and to enable them to digest with ease large amounts of food."

The reputation of the author and the conservative views herein set forth very strongly recommend this entertaining little book to the attention and confidence of the medical profession.

